# Irreducible Traumatic Radial Head Dislocation Due to Annular Ligament Interposition in a Child with Ulnar Plastic Deformation: A Case Report

**DOI:** 10.1111/os.12981

**Published:** 2021-05-04

**Authors:** Dung T Tran, Nam T Vu, Quynh T Nguyen, Toan D Duong, Du G Hoang, Son N Dinh, Son M Le, Thanh X Dao, Long H Nguyen

**Affiliations:** ^1^ Hanoi Medical University Hanoi Vietnam; ^2^ Saint Paul University Hospital Hanoi Vietnam; ^3^ Bachmai University Hospital Hanoi Vietnam; ^4^ VietDuc University Hospital Hanoi Vietnam

**Keywords:** Annular ligament, Dislocation, Elbow, Irreducible radial head dislocation

## Abstract

**Background:**

The traumatic dislocation of the radial head in children is commonly treated by closed reduction. Sometimes, however, this strategy of treatment may not be effective due to the location of soft tissues in the radio‐shoulder joint. The literature presents a few cases of the irreducible radial head dislocation with ulnar plastic deformation. Because it is a relatively rare condition, such a traumatic dislocation can be easily missed. Neglected injuries can lead to unwanted complications and unpredictable surgical outcomes.

**Case presentation:**

This study presents a relatively rare case of traumatic radial head dislocation with ulnar plastic deformation in a 3‐year‐old child, which was successfully treated by open reduction. The examined case did not require osteotomy and ligamentous reconstruction. The initial attempt of closed reduction failed due to annular ligament interposition, which has been detected on MRI. After 3 months of treatment, the range of motion of the operated arm gradually improved. At the 6‐month follow‐up, the Mayo elbow‐performance score indicated an excellent treatment outcome.

**Conclusions:**

The delayed treatment of radial head dislocation with ulnar plastic deformation can hinder the supination and pronation of the forearm, resulting in elbow/forearm deformity. The earlier this condition is detected, the easier it will be to treat it and the better the treatment outcome will be. The examined case of irreversible traumatic dislocation, successfully treated by open reduction, may help to treat radial head dislocation better.

## Graphical abstract

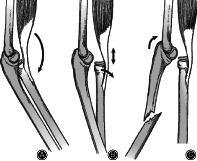

Traumatic radial head dislocation with ulnar plastic deformation is very rare. These neglected traumas resulted in a far more complex injury. Here is a rare case of injury in a 3‐year‐old child that was successfully treated. The initial close‐reduction attempt was fail due to annular ligament interposition. Delayed treatment can lead to deformity of elbow and forearm.

## Introduction

The physician Monteggia first described a case of a diaphyseal fracture in the forearm ulna with the concomitant dislocation of the head in 1814. This type of fracture dislocation is seen in 0.6%–2.0% of children. It is a very rare forearm injury, accounting for 2.3% of all forearm injuries^1^. That said, the results of the corresponding treatment are disappointing since almost half (46.3%) of the patients fail to heal^2^. This makes it crucial to establish an effective way to treat ulna shaft fractures.

If the Monteggia fracture is fresh, it can be treated by using the closed reduction strategy. At the same time, the chronic cases of fracture dislocation are harder to treat. Due to the presence of anatomical and functional disorders in the proximal part of the forearm and brachioradial joint, the subsequent treatment may not restore the biomechanical properties of the elbow joint^3^. Without treatment, the scarring of the wound canal and the retraction of the interosseous membrane and biceps head can be seen within 8–15 days after the injury^4^. Consequently, it may not be possible to set the dislocated humeral head effectively and in time.

The main reason for ulna fracture complications is misdiagnosis^5^. During the acute phase of the injury, physicians are not able to detect the radial head dislocation. As a result, the patient undergoes treatment for a radial head fracture. The complications that may develop due to such a mistreatment, according to some data, account for 12%–50% of the cases^6^. The most frequent complications include the early post‐traumatic arthrosis and contracture (62%–82% of the cases) and heterotopic ossification (14% of the cases)^7^. Based on the above, the treatment of ulna fractures with the concomitant head dislocation, especially in children, is an important and urgent problem in medicine. With positive ulnar variance (ulna+), the injury to the distal radioulnar joint may facilitate degenerative changes with the fibrocartilage complex, resulting in ulnocarpal impingement. In addition, 19% of children with ulnar fractures have pain in the wrist joint, and 16% have a significantly limited range of movement in the wrist joint^8^. Other consequences include abnormal adhesions (9%), deformities (15%), contractures (14%), ankylosis (2%), neurovascular disorders (2%), osteomyelitis (2%), and false joints (1%)^9^. Therefore, the long‐term implication of ulnar fracture dislocation is disability, the severity of which depends on the severity of the injury. Factors that influence the successful treatment outcomes include the time of patient enrollment, the use of new treatments, and trauma research^4^.

Traumatic radial head dislocation with ulnar plastic deformation is a rare injury^10^, ^11^. It can be detected if one carefully evaluates the radiographs of an injured elbow, taking into account the radiocapitellar line and the ulnar bow^12^. Such an irreducible traumatic dislocation can be easily missed and whose later detections are often diagnosed as a neglected Monteggia fracture^13^, ^14^, ^15^. The rate of missed injuries has been as high as 50%^16^. It is difficult to reduce a radial head dislocation with ulnar plastic deformation by closed manipulation, unless the procedure is performed immediately after injury^17^. In this case, surgical treatment is required, but it is difficult and complex, therefore additional methods are needed, such as ligament reconstruction or osteotomy. In case of timely diagnosis, the dislocation of the dislocated radial head is easily achieved by traction. However, sometimes the location of the soft tissue in the radial joint prevents this^18^. In children, bowing of a long bone is common due to increased plasticity^16^.

The most commonly reported factors are annular ligament and anterior capsules; however, others include the biceps tendon, the median nerve radial nerve, osteochondral bone fragments, *etc*.^19^, ^20^, ^21^, ^22^, ^23^, ^24^, ^25^. We herein introduce a case of radial head dislocation, which was detected 5 days after the initial trauma and treated by open reduction, repair of the annular ligament, with no need for osteotomy or joint fixation, and resulted in an excellent outcome.

## Case Presentation

A 3‐year‐old girl was referred to our hospital 5 days after the trauma, complaining of right elbow joint restriction and pain. She fell onto her outstretched hand while playing at home from a low chair. Her hand was put into a sling by her grandmother, but she hadn't received any other medical care.

On our examination, there was mild swelling over the elbow and tenderness over the radial head. On palpation, the radial head appeared to be anteriorly dislocated. She had pain in her elbow and could not do the active motion. The passive movement of the injured elbow was: 100º of flexion, 20º lack of full extension, 70º of pronation, and 50º of supination.

### Intervention

**Fig. 1:**
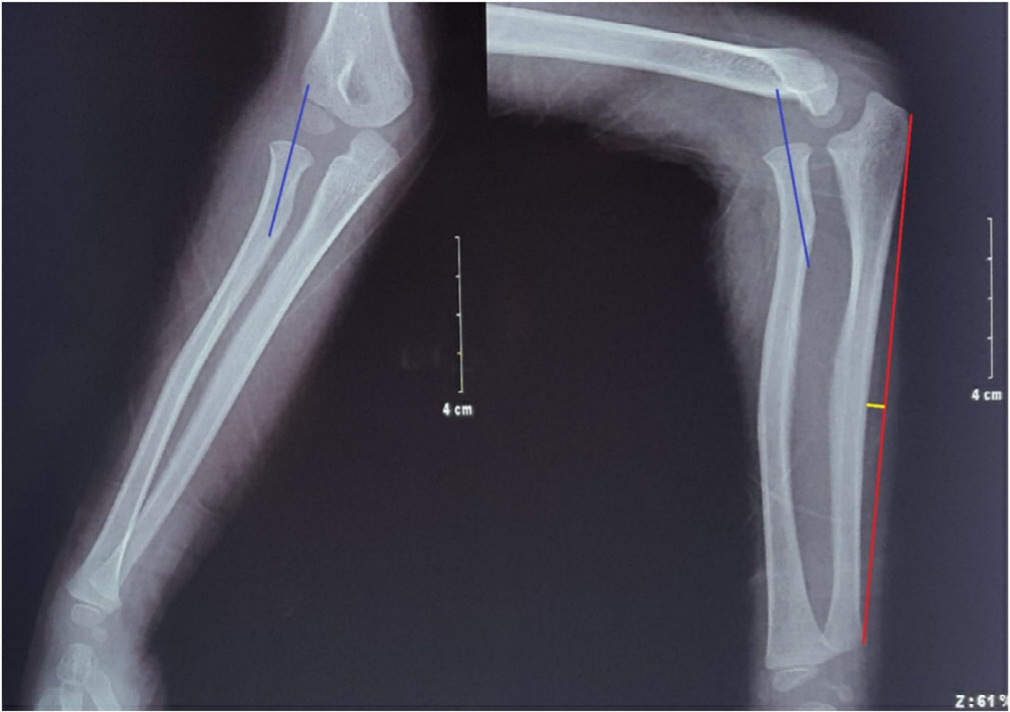
Preoperative X‐ray: The height of the bow‐shaped ulna is 5 mm (yellow line).

**Fig. 2:**
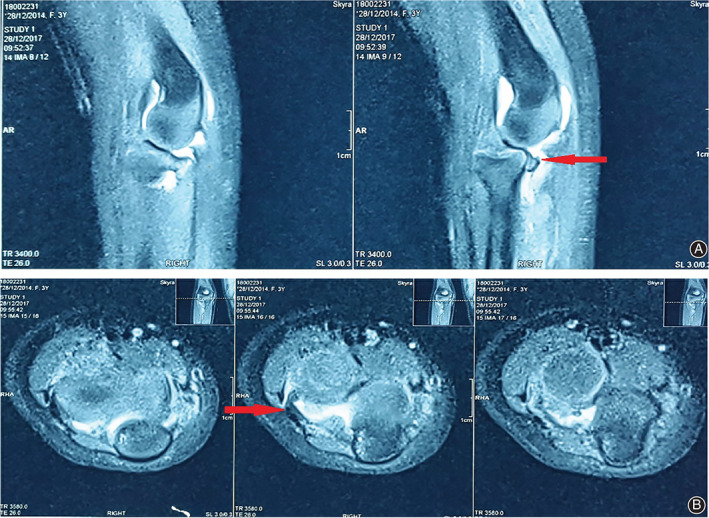
Preoperative MRI scans, red arrow: the annular ligament stuck in radiohumeral joint. A, side view; B, front view.

Radiographs confirmed an anterior dislocation of the radial head with plastic bowing of the ulna, no evidence of fracture (Fig. 1). Ultrasound detected fluid in the joint and soft tissue interposed in the humeroradial joint, so we anticipated that closed reduction would be unsuccessful. The patient was brought to the imaging department the following day. Under anesthesia using a laryngeal mask airway, closed reduction was initially attempted; however, that was unsuccessful as we anticipated. Then, a magnetic resonance imaging (MRI) film was done that showed that a structure of low signal intensity (annular ligament) was stuck in the humeroradial joint (Fig. 2).

**Fig. 3:**
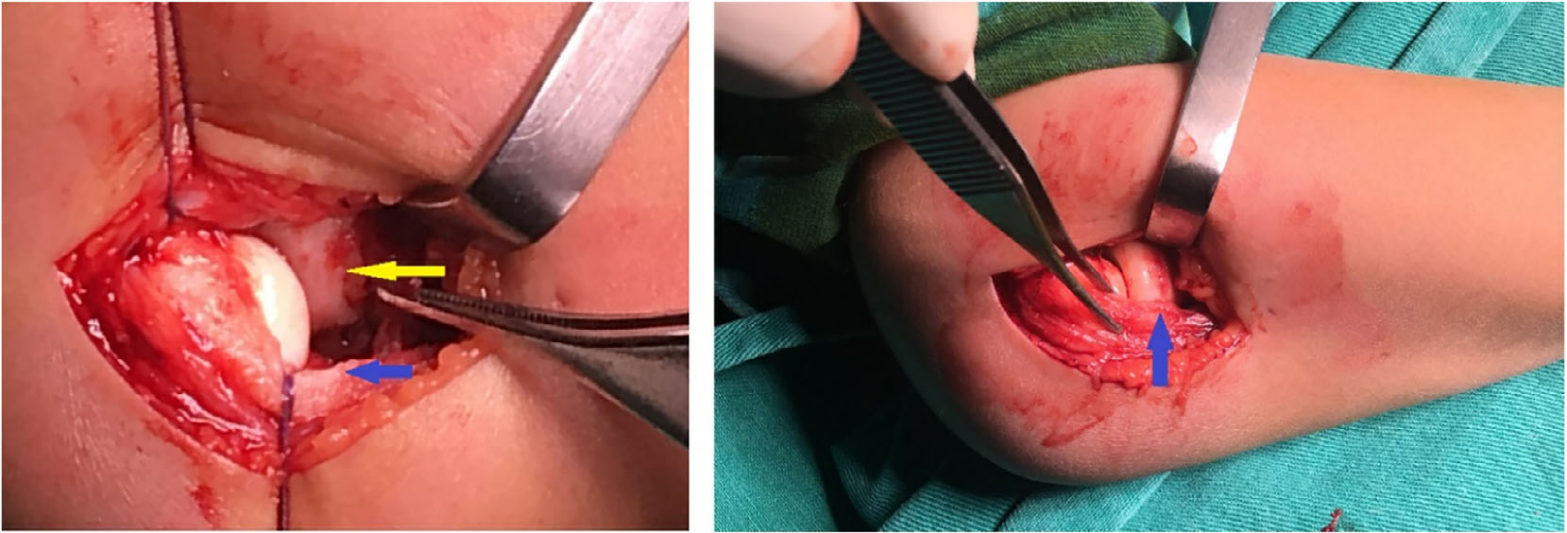
Intraoperative photograph. Yellow arrow: The annular ligament stuck in the humeroradial joint. Blue arrow: The annular ligament was transected posterior.

The patient was moved to the operative room the following day (7 days after injury). Closed reduction was attempted a second time, but it also proved unsuccessful verified by intraoperative X‐ray fluoroscopy. Open reduction through a posterolateral approach was performed. We found the annular ligament was what stopped the radial head from returning to its anatomical location. The annular ligament wasn't damaged but was very stretched. The radial head was found to be protruding below the annular ligament and was incarcerated between the ligament and anterior capsule, similar to a kind of buttonhole effect (Fig. 3). The ligament could not be pulled over the radial head when still intact as that made the joint irreducible. We decided to transect the ligament and repaired it subsequently. By pushing the radial head distally and pulling the interposed ligament anteriorly, it returned to its normal anatomical position immediately. After reduction, the elbow seems to be stable in the proper position during elbow joint movement and forearm rotation, so we finished the operation.

**Fig. 4:**
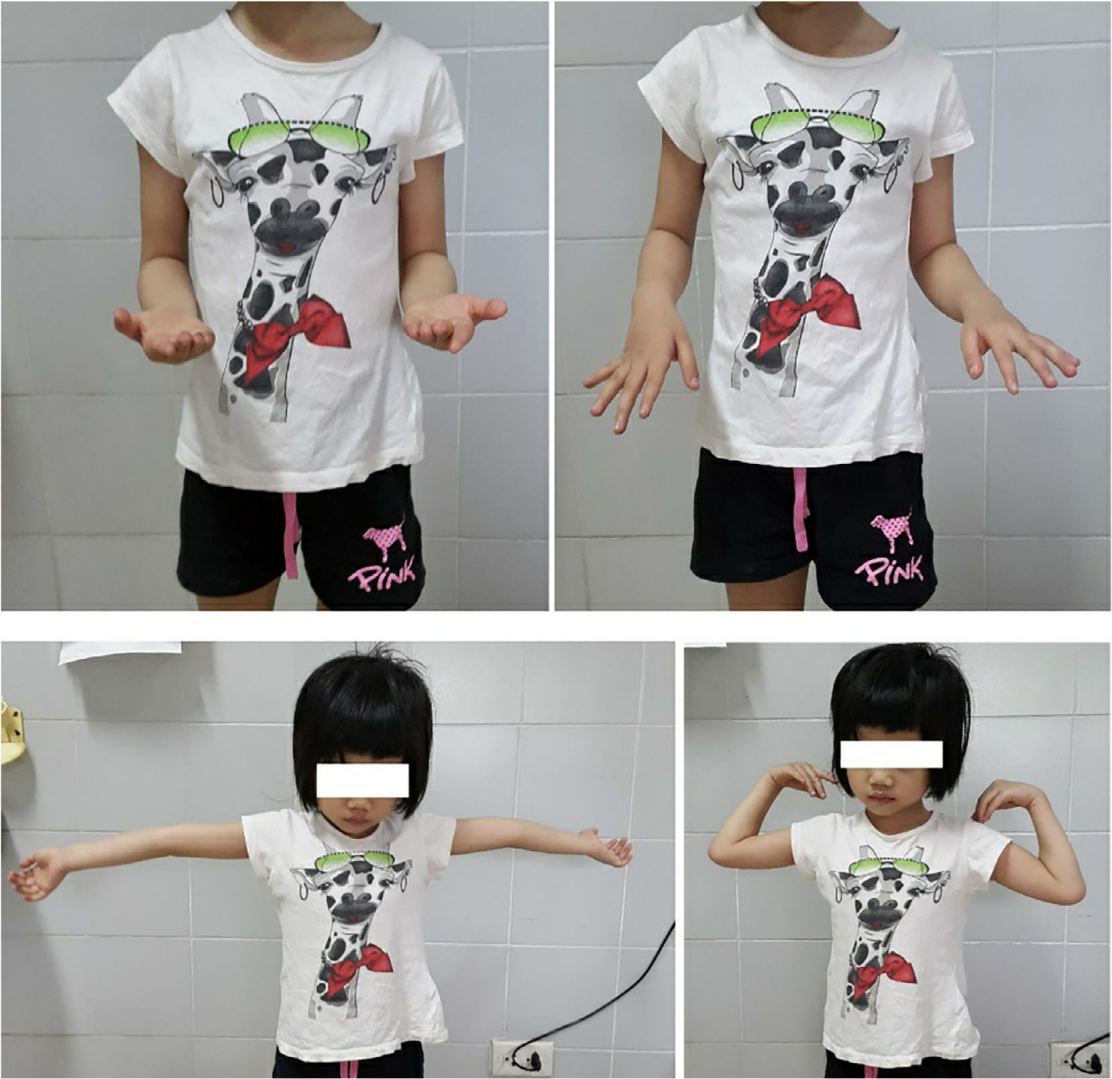
The 6‐month postoperative range of motion.

Active range‐of‐motion exercises were allowed following 3 weeks of immobilization by a long arm plaster splint. At 3‐week, 6‐week, 3‐month follow‐up checkups, the movement range of the operated arm gradually improved, and at 6 months after surgery, it was 135º of flexion, full of extension, 90º of pronation, 90º of supination. This was compared to normal, which was 145º of flexion, full of extension, 90º of pronation, 90º of supination (Fig. 4). The result was excellent according to the Mayo elbow performance score (100 scores)^26^.

## Discussion

Although it has been reported in the literature, acute traumatic radial head dislocation with ulnar plastic deformation in children has not been considered nor indicated appropriate treatment. Many cases were missed or misdiagnosed, and later detection was reported as “old radial head dislocation” or “neglected Monteggia fracture” with often more difficult and complex treatment but with limited results. This injury is classified by some authors as a subgroup of the Monteggia fracture. Kajiwara *et al*.^27^ classified the traumatic “isolated” radial head dislocation in Monteggia fracture‐dislocations type A fracture. It can be classified as Bado equivalent lesion type 1, which including “nursemaid's elbow,” also known as radial head subluxation in children Flynn *et al*.^18^. They are grouped together by the same anatomical lesion and mechanism that is related to the injury of the annular ligament trapped inside the joint.

It is pertinent to point out that the term “Monteggia fracture” refers to all fractures of the ulna, irrespective of the location, associated with dislocation of the radial head^28^, ^29^. If the fracture goes undetected and/or is not promptly treated, it may lead to ulnar malunion and persistent radial head dislocation with subsequent loss of function, pain, degenerative arthritis and, in some cases, late neuropathy^30^, ^31^. In our patient, plain radiographs showed an anterior medial dislocation of the radial head and a plastic bowing of the ulna so it could be classified as Bado type I equivalent or Letts type. According to the four‐tier sub‐classification based on the anatomical variation of the fracture dislocation, Type 1 refers to a proximal diaphyseal ulnar fracture with anterior dislocation of the radial head^32^.

The closed reduction failed. In Monteggia lesions, if the fracture of the ulna is anatomically reduced even by a closed or open procedure, the reduction of the dislocated radial head should be easily achieved without additional incision^33^. However, there are a few cases in which reduction of the dislocation cannot be accomplished by a closed procedure because the interposition of soft tissues in the radiohumeral joint obstructed the reduction. Other authors reported the most common interposed factors were annular ligament and anterior capsules^13^, ^14^, ^15^. In our case, it was an annular ligament, which was confirmed on both MRI films and intra‐operative expose.

Triantafyllou *et al*.^19^ reported a case of an unsuccessful closed reduction in a 5‐year‐old boy with a “pulled elbow,” a type of radial head dislocation, complicated with a transverse tear in the annular ligament. Takas and Mizuochi^20^ described a case of a 6‐year‐old girl with irreducible dislocation of the radial head and undisplaced olecranon fracture in which the radial head was found to be protruding through a buttonhole tear of the anterior joint capsule, precluding closed reduction. Bradley *et al*.^21^ presented the case of a 5‐year‐old girl in whom an open procedure was required for the reduction of the dislocated radial head. The irreducibility was shown to be caused by the annular ligament that had slipped posterior to the radiocapitellar joint and was compressed between the radial neck and capsule. Neviaser and LeFevre^22^ reported the case of a 7‐year‐old boy with a dislocated radial head, protruded through a transverse tear capsule, with no damage to the annular ligament. Other reported factors causing irreducible dislocations include the biceps tendon, the median nerve radial nerve, osteochondral bone fragments, etc., interposed in the joint space^15^, ^23^, ^24^, ^25^. Some authors reported finding the factors with MRI scans. O'Neill *et al*.^26^ and Kajiwara *et al*.^27^ described irreducible pulled elbows in an adolescent and an adult, respectively, in which plain radiographs did not demonstrate any bony anomaly around the elbow joint; however, MRI films showed a subtotal anterior subluxation of the radial head and the annular ligament trapped in the radiohumeral joint. The advantages of MRI scans are that they help us to confirm the diagnosis, to investigate the interposed factors, and to anticipate a probable surgical treatment. The disadvantages are MRI scanners can be affected by children's movement, which is also the reason why we attempt to reduce the dislocation of our patient in the imaging department under anesthesia using a laryngeal mask airway.

Our patient was treated successfully by open reduction and repair of the annular ligament. However, if she wasn't promptly diagnosed, it would be necessary to do other more difficult surgery. In neglected radial head dislocation, the annular ligaments trapped inside joints under compress loading would be sclerosed or necrotic; therefore, despite releasing and reducing the radial head, the ligament has also been ineffective. Indications for annular ligament reconstruction has still many controversies, but we support Bhaskar's view^7^: the decision depends on the results of the stability of the radial head intraoperatively.

Furthermore, there is a bone‐length discrepancy between the radial and ulna problem due to the plastic deformation of the ulna. Long bones with curve deformation in children was described by Borden^34^ in 1974 as caused by flexibility in their bones, thick periosteum, and partially natural curvature of bone; it results when the axial compression force is applied to a curved bone exceeds the bone's elasticity, but is below the fracture threshold. This curvature persists even after removal of the force. In our opinion, in cases of acute injury without soft tissues interposed, such as stretching the elbow joint, the ulna may be elastic enough to reduce curvature and the radial head could return to its original location. However, the neglected lesion has to be treated similarly to a neglected Monteggia fracture with an indication of the ulnar osteotomy. Many authors used the 4‐week time after injury to determine whether chronic radial head dislocation had occurred^14^, ^34^, ^35^.

Sai^14^ in 2005 reported five cases of isolated radial head dislocation in children: two cases 4 days after injury were successful with closed reduction, two cases 14 days after injury and 19 days after injury were treated by open surgery for reduction and repair of the disrupted annular ligament. The remaining case was 62 days after injury, and was carried out with ulnar osteotomy. Takase and Mizuochi^20^, Bradley *et al*.^21^, and Chung *et al*.^36^ reported cases treated successfully with open reduction, and their patients came for surgery 1 day, 3 days, and 30 days after the initial trauma, respectively. Gupta *et al*.^37^ performed an open reduction of the radial head and reconstruction of the annular ligament on cases with a duration of delayed diagnosis of 6 weeks and 3 months. Singh and Kumar^38^ reported threes cases with an average duration of delayed presentation of 8 to 13 weeks from the initial injury, and all cases underwent ulnar osteotomy.

Iirreducible radial head dislocation with ulnar plastic deformation is not reported a lot in the literature. However, the sooner the detection is, the simpler the treatment is, and, the better the result is. We, therefore, present a rare case of irreducible traumatic radial head dislocation and ulnar plastic bowing deformity was treated successfully with open reduction but need neither osteotomy nor annular ligament reconstruction.

## Authorship declaration

All authors listed meet the authorship criteria according to the latest guidelines of the International Committee of Medical Journal Editors. All authors are in agreement with the manuscript.

This research has been approved by the IRB of the authors' affiliated institutions and by the Ethics Committee of Hanoi Medical University Hospital.
